# Chemoembolization With Drug-Eluting Beads for the Treatment of Hepatocellular Carcinoma

**DOI:** 10.6004/jadpro.2016.7.7.8

**Published:** 2016-11-01

**Authors:** Kathy Diener Dasse, Michael J. Lander, Paula M. Novelli

**Affiliations:** 1 Department of Pharmacy Services, University of Michigan Health Systems, Ann Arbor, Michigan;; 2 Pharmacy Services, Mayo Clinic – St. Mary’s Hospital, Rochester, Minnesota;; 3 Division of Vascular and Interventional Radiology, University of Pittsburgh Medical Center, Pittsburgh, Pennsylvania

**ABSTRACT**

**CASE STUDY**

THA is a 40-year-old immigrant from Myanmar who has been living in the United States for about 6 years. He has a history of hepatitis B virus (HBV) infection for many years, for which he has never received any treatment. He is negative for hepatitis B ’e’ antigen (HBeAG), with normal liver enzymes on his routine primary care visits. 

THA was referred to a hepatology clinic when recent laboratory studies revealed a slowly increasing tumor marker—alpha-fetoprotein level (AFP). His AFP increased from a normal level of 8 ng/mL (normal range, 0–8 ng/mL) in December 2014 to 44 ng/mL in June 2015. His HBV DNA level was 30,409 IU/mL. Because of his history of chronic hepatitis B infection and the rising AFP level, a computed tomography (CT) scan was done and showed a 4 cm × 3.5 cm liver mass in segment 8, accompanied by cirrhotic morphology to the liver (see Figure 1A–Figure 1D on page 765).

As THA has no other medical problems and remains active in his community and at home, he was referred to a liver cancer treatment program for consideration of liver transplantation, which could be an effective cure. THA is classified as Barcelona Clinic Liver Cancer (BCLC) A (early disease, single tumor) with an Eastern Cooperative Oncology Group (ECOG) performance status of 0, Child-Turcotte-Pugh A cirrhosis.

THA was determined to be a good candidate for transplant, based on the BCLC and Milan criteria (Forner, Reig, de Lope, & Bruix, 2010; Mazzaferro et al., 1996). The model for end-stage liver disease (MELD) score was 22 for hepatocellular carcinoma, based on the Organ Procurement and Transplantation Network (OPTN)/United Network for Organ Sharing (UNOS) guidelines (which are followed by our institution, University of Michigan Health System, for liver allocation). His tumor met the size criteria for a good outcome after transplant, and he had the necessary social support, as transplantation is a lifelong event requiring both social and psychological adjustment.

THA was then referred to interventional radiology for consideration of locoregional liver-directed therapies. This would offer disease control and stability while he underwent the additional testing necessary before being placed on the transplant list. After a patient is formally placed on the list for transplant, there is at least a 6-month wait to receive a liver due to the organ allocation process and medical necessity. Bridging liver-directed therapies protect a patient’s candidacy for curative transplant and may help to decrease the dropout rate from the transplant waiting list, thereby having a positive effect on posttransplant survival and tumor recurrence rates (Graziadei et al., 2003; National Comprehensive Cancer Network [NCCN], 2015).

During his transarterial chemoembolization procedure, THA underwent an arteriogram, which revealed the solitary hypervascular mass in the right lobe. Selective transarterial chemoembolization was then done by injecting 1 vial of 100 to 300 µm drug-eluting LC Bead particles loaded with 75 mg of doxorubicin into the target artery supplying the tumor. A postembolization arteriogram showed no residual tumor blush (see Figure 1E below).

THA experienced mild nausea and several days of minimal fatigue. He recovered well, with no residual effects. A follow-up magnetic resonance imaging performed 6 weeks after his procedure revealed greater than 95% tumor response (see Figure 1F below). THA now will undergo imaging every 6 weeks until he receives a transplant.

**Figure 1:**
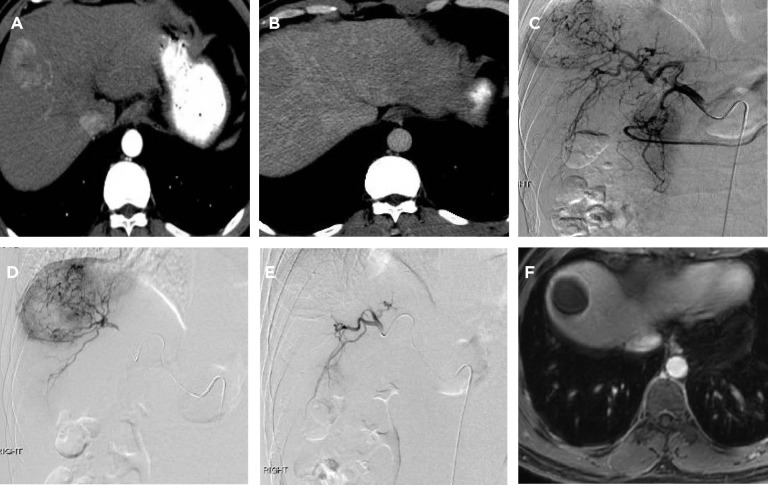
THA’s scans. (A) Arterial-phase CT scan shows the 4 cm mass in the liver; (B) Delayedphase CT scan of the liver shows the "washout" of contrast typical for hepatocellular carcinoma; (C) Right hepatic arteriogram shows the tumor vascularity in the dome of the liver; (D) Selective segmental right hepatic arteriogram shows the tumor vascularity; (E) Postembolization right hepatic arteriogram shows no further filling of the tumor; (F) Contrast-enhanced arterial-phase MR imaging 6 weeks after DEB-TACE shows no residual tumor vascularity.

**ARTICLE**

Primary liver cancer is one of the most common malignancies occurring worldwide as well as one of the most common causes of cancer-related deaths (American Cancer Society [ACS], 2015; El-Serag & Rudolph, 2007; Torre et al., 2015). Hepatocellular carcinoma (HCC) accounts for approximately 90% of primary liver cancer. Incidence and mortality vary based on geography, race or ethnicity, and gender. In 2012, there were an estimated 782,500 new cases of liver cancer worldwide and 745,500 deaths. More recent statistics for the United States were an estimated 35,660 new cases of liver cancer, with approximately 75% being hepatocellular carcinoma, and 24,550 deaths.

Worldwide, chronic infection with hepatitis C virus (HCV) and hepatitis B virus (HBV) is the primary risk factors for HCC. As was the case with our patient THA, as many as one in eight individuals are chronically infected with HBV in countries such as Myanmar (Custer et al., 2004). Obesity, diabetes, liver disease associated with alcohol use, and smoking tobacco, along with chronic infection due to HBV and/or HCV, are major risk factors for liver cancer in the United States (ACS, 2015). To further emphasize the differences in risk factors among various parts of the world, diabetes and/or obesity account for approximately one-third of all primary liver cancers, and alcohol use/abuse accounts for approximately one-quarter of primary liver cancers in men in the United States (ACS, 2015; El-Serag & Rudolph, 2007).

A separate risk factor, genetic hemochromatosis, is due to excess iron absorption related to mutations in the high iron (HFE) gene (NCCN, 2015). Although this genetic defect is relatively rare, affecting between 1 in 200 and 1 in 400 persons of Northern European ethnicity, those with cirrhosis and hereditary hemochromatosis have a 20- to 200-fold increase in lifetime risk of HCC (Harrison & Bacon, 2005).

Treatment strategies and survival depend on various disease and patient factors. When used early in the course of their disease, surgical therapies such as resection or liver transplantation and ablative treatments such as percutaneous local radiofrequency ablation and ethanol injections yield a 5-year survival rate of 50% to 70% (Bolondi et al., 2012). Despite the overall 5-year survival being similar for both liver resection and transplantation in patients with early disease, recurrence has been observed to be greater in patients treated with liver resection than with liver transplantation (Sapisochin et al., 2013). The cumulative risk of tumor recurrence at 1, 5, and 10 years was 18%, 69%, and 83% in patients treated with liver resection and 4%, 18%, and 20% in patients treated with transplant (*p* < .001), respectively. The 10-year actuarial survival for patients treated with resection was 33%, compared with 49% for patients who received a liver transplant (*p* = .002). Systemic therapy with sorafenib (Nexavar) in patients with advanced disease yields a 1-year survival rate of approximately 44% (Bolondi et al., 2012). Transarterial chemoembolization (TACE) is an option for those patients with unresectable HCC, localized disease, and adequate performance status and liver function, yielding a 1- to 2-year survival rate of 82% and 63%, respectively (Bolondi et al., 2012).

Chemoembolization combines an agent that occludes the hepatic artery feeding the tumor and local release of a chemotherapeutic agent. The rationale for this procedure is that embolization causes necrotic damage to the tumor, and systemic exposure to chemotherapy is limited (Lewandowski, Geschwind, Liapi, & Salem, 2011). An added benefit is that hypoxic damage to the tumor cells may facilitate the uptake of chemotherapy by those cells (Lewandowski et al., 2011; Kruskal et al., 1993). Tumor necrosis also reduces the risk for tumor dissemination during transplant surgery.

Although this article focuses on the treatment of HCC, other malignancies with metastatic disease isolated to the liver have also been treated with chemoembolization. For example, it has been used to treat liver metastases due to colorectal cancer, cholangiocarcinoma, neuroendocrine tumors, breast cancer, and melanoma (Agarwala, Eggermont, O’Day, & Zager, 2014; Bester, Meteling, Boshell, Chua, & Morris, 2014).

## STAGING AND TREATMENT DECISIONS

Multiple staging systems, based on various disease factors and liver function, are used to determine the course of therapy and prognosis in staging HCC. Following the initial diagnosis, the National Comprehensive Cancer Network (NCCN) categorizes patients into four categories based on the potential for success with various treatment strategies. They include: patients with disease that may be treated with surgery (either resection or transplantation) and whose performance status (PS) and comorbidities allow for such surgery; patients for whom surgery is not an option due to PS and/or comorbidities; patients with unresectable disease; and patients with metastatic disease (NCCN, 2015).

For patients with unresectable disease who are being treated with TACE, the NCCN refers to two staging systems (NCCN, 2015). One of the systems is the Cancer of the Liver Italian Program (CLIP; 1998). This system uses Child-Pugh stage, tumor morphology, alpha-fetoprotein (AFP), and portal vein thrombosis in scoring disease to aid in determining prognosis and planning treatment.

The CLIP system was compared with six other staging systems in patients who had undergone TACE to determine which was the most useful in this population (Cho et al., 2008). Multivariate analysis demonstrated that low serum albumin levels (≤ 3 g/dL), ascites, elevated serum AFP level (> 60 ng/mL), and portal or hepatic vein tumor thrombosis were significant risk factors for death (*p* = .001, *p* = .001, *p* = .004, *p* = .000, respectively). When survival statistics for each stage of disease according to the various systems were compared, the CLIP system had distinct survival periods without overlap, as well as a statistical difference between adjacent prognostic scores. These outcomes indicate the validity of this particular staging system.

**Figure 2:**
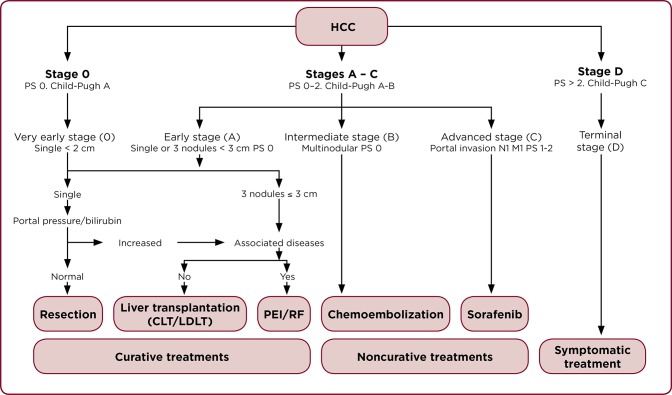
Barcelona Clinic Liver Cancer (BCLC) staging, including strategies for treatment of hepatocellular carcinoma (HCC). Chemoembolization is recommended for intermediate-stage HCC. Reproduced with permission from Forner et al. (2010). CLT = cadaveric liver transplant; LDLT = living donor liver transplant; PEI = percutaneous ethanol injection; PS = performance status; RF = radiofrequency.

The second staging system mentioned by the NCCN with regard to TACE for HCC is the Barcelona Clinic Liver Cancer (BCLC) staging system (NCCN, 2015; Forner, Reig, de Lope, & Bruix, 2010; see Figure 2). The BCLC system also considers multiple disease and patient characteristics in defining prognosis and treatment (Llovet, Bru, & Bruix, 1999). The BCLC points patients with multinodular disease and no extrahepatic spread or portal thrombosis, Child-Pugh A-B, and performance status of 0 (BCLC intermediate stage [B]) to treatment with chemoembolization (Llovet et al., 1999; Liccioni, Reig, & Bruix, 2014), with median survival being greater than 4 years in some studies (Burrel et al., 2012; Malagari et al., 2012).

Because of the heterogeneity of patients considered BCLC stage B, researchers have worked to further subdivide the class, with the goal of selecting an appropriate population for treatment with TACE (Bolondi et al., 2012; Ha et al., 2014). These subclassifications are based on liver function, performance status, and tumor burden. Median survival time following the first treatment with TACE differed between these subgroups (B1 subgroup [41 months] vs. B2 subgroup [22.1 months], *p* < .001; B2 subgroup [22.1 months] vs. B3 subgroup [14.1 months], p = .001; there was no difference between B3 [14.1 months] and B4 subgroups [17.2 months], p = .48; Ha et al., 2014).

Other groups have advocated offering treatment with TACE to an even broader population of patients with HCC. One such group is the Expert Panel Opinion on Interventions in Hepatocellular Carcinoma (EPOIHCC), which recommends expanding the appropriate patient population to include those with BCLC A, B, or C disease, Eastern Cooperative Oncology Group (ECOG) PS < 2, and Child-Pugh < C (Cheng et al., 2014). TACE has also been shown to be effective in patients recommended for liver transplantation if the expected time to transplant is more than 6 months (Cheng et al., 2014; Graziadei et al., 2003; Otto et al., 2007). Extensive extrahepatic disease is still considered a contraindication to treatment with TACE (Cheng et al., 2014).

The BCLC staging system (see Figure 2) links this patient to resection or transplantation for curative therapy. Liver transplant was chosen based on the patient’s age and underlying hepatitis B infection. Our patient, THA, benefited from locoregional therapy, in that treatment with drug-eluting beads–TACE allowed for control of his disease while workup for liver transplant took place. Furthermore, with this sort of bridging therapy, the wait for transplantation did not negatively affect his overall prognosis.

## CHEMOEMBOLIZATION

**Conventional TACE**

Conventional TACE is not a new technology. Use of embolizing agents mixed with chemotherapy or given alone (bland) for the treatment of HCC dates back to the late 1970s and 1980s (Talenfeld, Sista, & Madoff, 2014; Wheeler et al., 1979; Hirai et al., 1989; Sasaki et al., 1987; Patt et al., 1983). Materials used as embolizing agents have included gelatin particles, polyvinyl alcohol (PVA) particles, and ethiodized oil (Lipiodol).

Limitations to the use of some of these agents include large particle size or irregular shape, making it more likely larger vessels will be occluded instead of the smaller targeted distal vasculature feeding the tumor. Incomplete vascular occlusion is another issue with some of these materials, as well as problems related to clogging the catheter used to deliver the agent to the intended area of the vasculature (Tam, Leung, & Wang, 2011). Embolizing agents with a spherical shape and calibrated sizes are now available and may overcome some of the issues associated with the use of gelatin sponge and PVA particles (Osuga et al., 2012).

Ethiodized oil has also been used in combination with various chemotherapy agents, such as doxorubicin, epirubicin, mitoxantrone, fluorouracil (5-FU), mitomycin, and cisplatin, for the treatment of HCC. It allows for the delivery of the chemotherapy to the site of the tumor with subsequent prolonged, local release of the chemotherapy drug (Miura & Gamblin, 2015; Tam et al., 2011; Nakamura, Hashimoto, Oi, & Sawada, 1989). One issue with the use of ethiodized oil is incomplete arterial occlusion, making it necessary to add an embolizing agent (e.g., Gelfoam). Incompatibility with hydrophilic antineoplastic agents is another issue with the use of ethiodized oil.

To overcome compatibility issues, the chemotherapy drug must first be dissolved in other substances, such as water-soluble x-ray contrast agents. The mixture is then added to the ethiodized oil. This combination of chemotherapy, ethiodized oil and an embolizing agent, had been viewed as the standard of care for patients with intermediate-stage HCC until the introduction of drug-eluting beads (DEBs).

**Drug-Eluting Beads**

More recently, DEBs have emerged for the treatment of HCC. There currently are two DEB preparations available: LC Bead (also known as DC Bead outside the United States) and QuadraSphere Microspheres (also known as HepaSphere Microspheres outside the United States). The LC Beads are made of polyvinyl alcohol modified by sulfonate sodium salt, and QuadraSphere is made of vinyl alcohol and a sodium acrylate co-polymer. Both preparations consist of uniform-sized beads that can be loaded with various chemotherapeutic agents, most commonly doxorubicin, irinotecan, or oxaliplatin (Miura & Gamblin, 2015).

Pharmacokinetic studies of both preparations have been done in animal models to evaluate doxorubicin plasma concentrations, release rate, and concentration at the tumor site. The study that evaluated doxorubicin-loaded DEBs in a rabbit model of liver cancer demonstrated a significantly lower plasma concentration of doxorubicin compared with intra-arterial administration. A peak intratumor doxorubicin concentration of 413.5 nmol/g was observed at 3 days, compared with a peak tumor concentration of 0.09 nmol/g in the group receiving doxorubicin intra-arterially. Intratumor doxorubicin concentrations remained high through days 7 (116.7 nmol/g) and 14 (41.76 nmol/g) in the DEB group compared with the intra-arterial route (0 nmol/g within 1 hour of injection), intra-arterial followed by bland beads (5–25 nmol/g), or a combination of ethiodized oil, doxorubicin, and bland beads (12–36 nmol/g). Plasma concentrations in the group treated with DEBs were minimal at all measured time points (Hong et al., 2006).

Similar results were reported with the QuadraSphere preparation loaded with doxorubicin, with peak tumor concentrations being observed at 3 days (40.632–50.052 nmol/g) and detectable levels throughout the 7-day study period. Pronounced tumor necrosis was observed at 3 days and continued through the 7-day study period (Lee et al., 2010). Pharmacokinetic studies in patients were consistent with animal studies. There were significant differences in doxorubicin area under the curve (AUC) and Cmax (maximum concentration), with a better safety profile for DEB-TACE than for conventional TACE (Van Malenstein et al., 2011; Malagari et al., 2014; see Table 1).

**Table 1:**
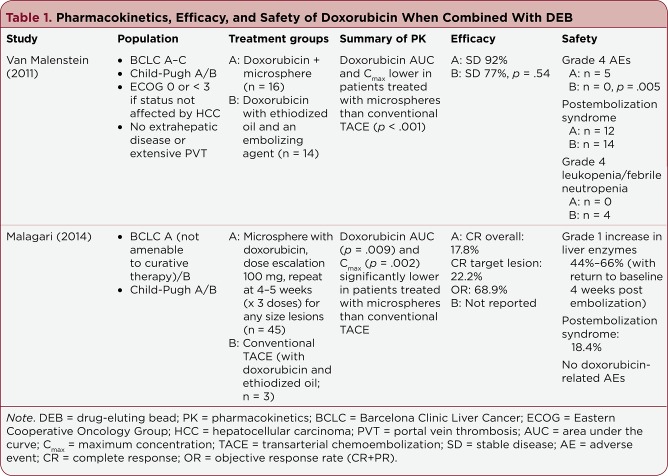
Pharmacokinetics, Efficacy, and Safety of Doxorubicin When Combined With DEB

Both bead preparations are available in various sizes to accommodate vessels of various sizes. LC Beads are available in 70–150 µm, 100–300 µm, 300–500 µm, and 500–700 µm sizes. QuadraSphere microspheres are supplied as dry particles, which swell when hydrated. The particle sizes of available QuadraSphere dry (hydrated) microspheres are 30–60 µm (120–240 µm), 50–100 µm(200–400 µm), 100–150 µm (400–600 µm), and 150–200 µm (600–800 µm). Studies have been conducted comparing various bead sizes to see whether there are differences in outcome depending on the bead size used.

One retrospective study conducted in patients with a diagnosis of HCC and Child-Pugh A/B, absence of portal vein thrombosis, no extrahepatic disease, and an ECOG PS of 0 to 2 compared a single TACE treatment using doxorubicin-loaded LC Beads with a particle size of 100–300 µm (n = 39) and 300–500 µm (n = 22; Padia et al., 2013). Choice of bead size was based on individual radiologist preference.

There was found to be a lower incidence of postembolization syndrome and fatigue after treatment in the 100–300 µm group (8% and 36%) compared with the 300–500 µm group (40 and 70%; p = .011 and p = .025, respectively). Complete response tended to be higher in the 100–300 µm group (59% vs. 36%, p = .114) and partial response was higher in the 300–500 µm group (8% vs. 27.3%, p = .055). The authors noted that the 300–500 µm size was likely chosen in some earlier clinical trials because it was similar to that of the embolization materials used in conventional TACE (Padia et al., 2013). Smaller-sized DEBs and the delivery of these beads closer to the tumor target may provide benefit due to smaller areas of ischemia induction and reduced ischemia in nontarget, normal liver tissue.

A second retrospective study showed similar results (Prajapati et al., 2014). This study included patients with BCLC advanced-stage disease, and the study allowed retreatment with DEB-TACE. Doxorubicin-loaded 100–300 µm beads (n = 59) were compared with a mixture of 300–500 and 500–700 µm (n = 35) LC Bead. Median overall survival was longer in the 100–300 µm bead size group (15.1 vs. 11.1 months, p = .005). Common terminology criteria for adverse events (CTCAE), grade III adverse events, and 30-day mortality were significantly lower in patients who were treated with the smaller beads (6.8% vs. 20%, *p* = .04 and 0% vs. 14.3%, p = .001, respectively). Again, there was found to be an advantage in the use of smaller-sized beads for DEB-TACE.

Our patient (THA) was treated with LC Bead 100–300 µm loaded with 75 mg of doxorubicin. He suffered minimal adverse events, primarily several days of mild fatigue and some mild nausea. Our institution uses only LC Bead 100–300 µm, although the other sizes are on the formulary.

When the QuadraSphere microsphere preparation was first marketed, our institution considered adding the product to our formulary. Because of the absence of studies directly comparing the two bead preparations and existence at that time of extended stability data only for the doxorubicin-loaded LC Bead preparation (Hecq et al., 2013), the QuadraSphere microsphere preparation was not added to our institution’s formulary. Risk for use of the wrong bead preparation was also considered in the decision to carry only a single bead product. Since that time, the manufacturer of QuadraSphere has included extended stability information in its product information (Merit Medical Systems, 2013).

**Clinical Evidence on Drug-Eluting Bead Transarterial Chemoembolization**

Both prospective and retrospective studies have compared conventional TACE and DEB-TACE. Results of these studies demonstrated an advantage to DEB-TACE with regard to efficacy, toxicity, or both (see Table 2).

**Table 2:**
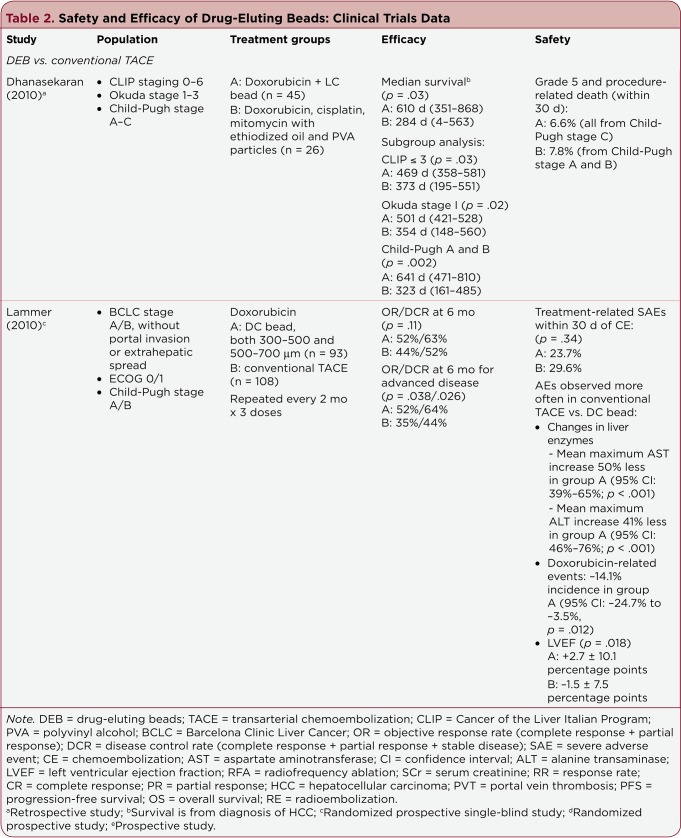
Safety and Efficacy of Drug-Eluting Beads: Clinical Trials Data

**Table 2 (cont.):**
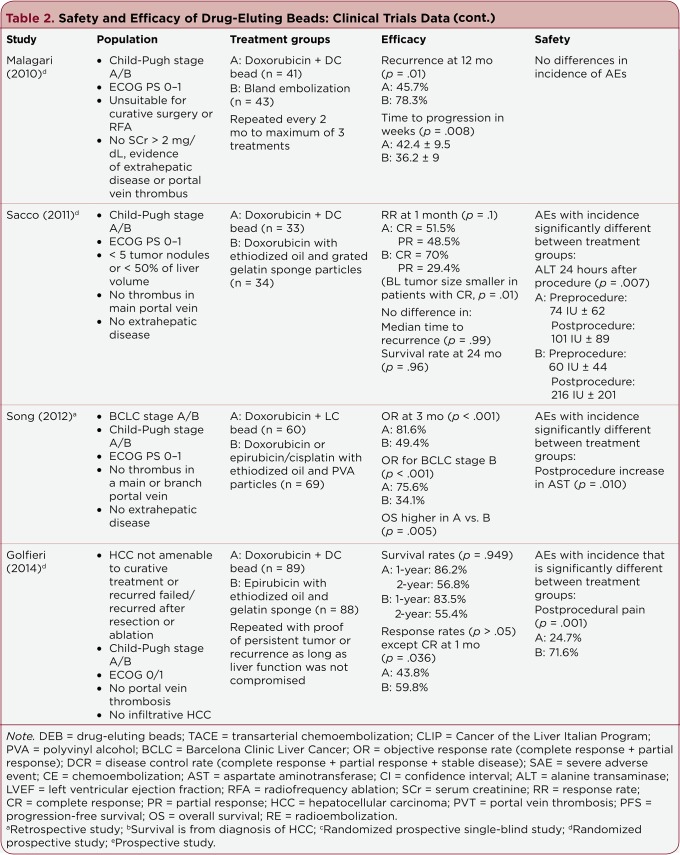
Safety and Efficacy of Drug-Eluting Beads: Clinical Trials Data (cont.)

**Table 2(cont.):**
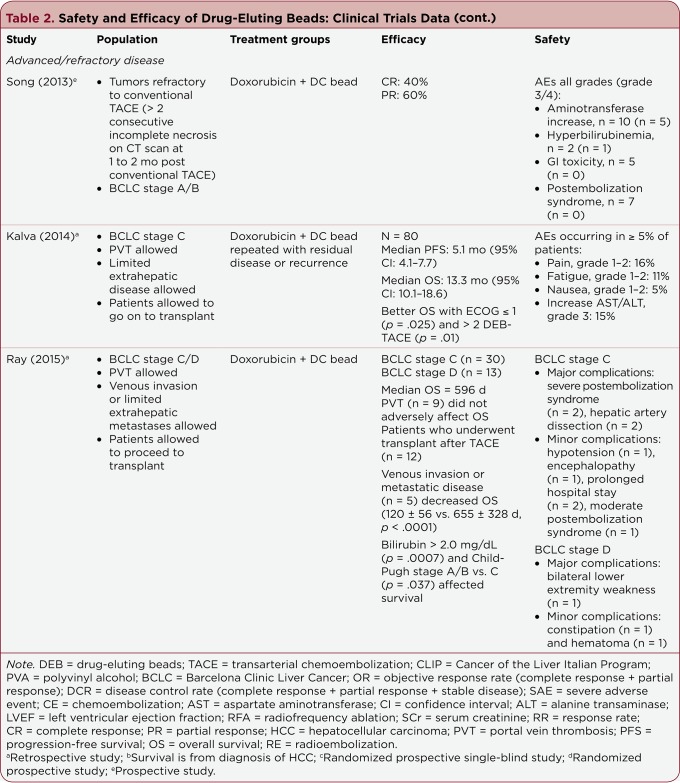
Safety and Efficacy of Drug-Eluting Beads: Clinical Trials Data (cont.)

**Table 2 (cont.):**
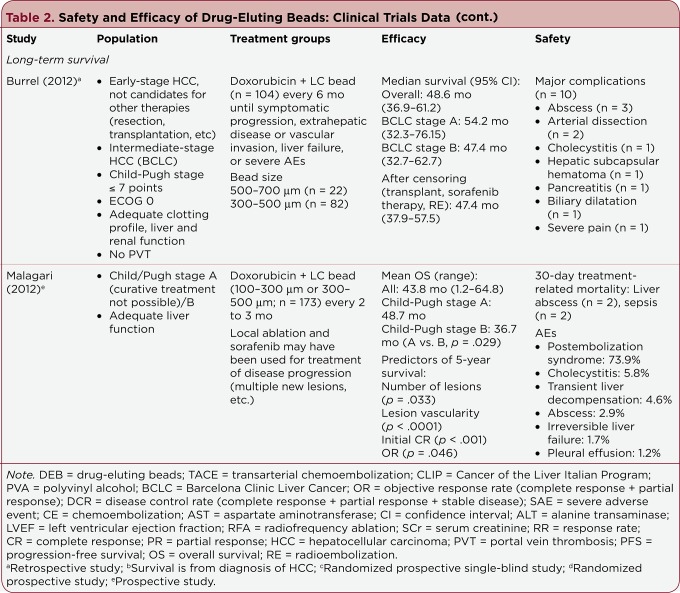
Safety and Efficacy of Drug-Eluting Beads: Clinical Trials Data(cont.)

One randomized, prospective, multicenter trial compared treatment with doxorubicin-loaded DC Bead (n = 93) to treatment with conventional TACE, which consisted of doxorubicin in ethiodized oil followed by an embolic agent administered every 2 months for a maximum of 3 doses (Lammer et al., 2010). At 6 months, objective response (OR) and disease control rates (DCR) did not differ significantly with DEB-TACE and conventional TACE (OR: 51.6% vs. 43.5%, *p* = .11; DCR: 63.4% vs. 51.9%, *p* = .11).

Supplementary analyses demonstrated differences in OR and DCR among patients with advanced disease (66.7% of patients in both groups), defined as Child-Pugh B, ECOG PS of 1, bilobar or recurrent disease (OR: 52.4% vs. 34.7%, p = .038; DCR: 63.5% vs. 44.4%, p = .026). No difference between groups in overall incidence of treatment-related serious adverse events was observed within 30 days of treatment (≤ = .34). However, there were fewer doxorubicin-related adverse events and less liver toxicity in the group treated with DEB-TACE compared with conventional TACE (*p* = .012, *p* < .001, respectively), despite a higher mean total dose of doxorubicin in the DEB-TACE group (295 vs. 223 mg).

With encouraging outcomes in patients with BCLC intermediate-stage HCC, there has been interest in treating patients with refractory or advanced HCC with DEB-TACE. One such study treated 10 consecutive patients with 10 tumors deemed refractory to conventional TACE of a total of 435 patients who had undergone TACE at a single institution. Refractory was defined as tumors that demonstrated more than two consecutive incomplete necroses on computed tomography (CT).

A complete response was observed in four of the tumors treated with DEB-TACE, and six patients had a partial response. Five patients were reported to have grade 3/4 aminotransferase elevations, and one patient was reported to have grade 3 hyperbilirubinemia, all of which returned to baseline within several days of treatment. Seven patients were reported to have grade 1/2 postembolization syndrome. The authors hypothesized that not all tumors retain ethiodized oil–doxorubicin, likely making conventional TACE less effective than the prolonged release of doxorubicin when given as DEB (Song et al., 2013).

Use of DEB-TACE has also been evaluated in patients with advanced disease. One retrospective study reported outcomes of DEB-TACE in patients with BCLC stage C (n = 30)/D (n = 13) disease (Ray et al., 2015). Median overall survival was 596 days. Predictors of poor outcome included gross vascular invasion or metastatic disease compared with no vascular invasion or metastases (overall survival: 120 ± 56 vs. 655 ± 328 days, *p* < .0001); bilirubin > 2.0 mg/dL (*p* = .0007); and Child-Pugh A vs. B/C (*p* = .037).

A second study conducted in patients with BCLC stage C disease demonstrated better outcomes in patients with an ECOG PS of ≤ 1 (*p* = .025) and patients who had more than two DEB-TACE procedures (*p* = .01; Kalva et al., 2014). Thus, DEB-TACE could be an option for certain patients with advanced-stage HCC.

Both authors noted that treating these patients with advanced disease with a combination of TACE and an antiangiogenic agent, such as sorafenib, would likely be beneficial. The rationale for the use of the combination centers on the hypoxic damage caused by TACE, which increases various angiogenic growth factors, such as vascular endothelial growth factor (NCCN, 2015; Sacco et al., 2015; Cabibbo et al., 2014). Sorafenib could be added to TACE to block the effect of these growth factors on tumor growth. Studies evaluating this combination are ongoing.

## ADVERSE EVENTS AND SUPPORTIVE CARE

Many of the studies in Table 2 demonstrated a better adverse-event profile with the use of DEB-TACE than conventional TACE. Differences in the rates of doxorubicin-related adverse events, changes in liver enzymes, and postprocedural pain have been observed (Lammer et al., 2010; Sacco et al., 2011; Song et al., 2012; Golfieri et al., 2014).

Postembolization syndrome is commonly reported in patients treated with either DEB-TACE or conventional TACE. Some authors noted that up to 80% of patients may exhibit one or more of the adverse events associated with postembolization syndrome, which can include abdominal pain, nausea, fever, fatigue, and transient increases in liver enzymes (Miura & Gamblin, 2015). These symptoms are self-limiting and rarely a cause for patient or practitioner concern. Other more serious, rare (< 1%) adverse events related to the procedure include hepatic abscesses, biliary sclerosis, liver failure, ischemic cholecystitis, vascular injury, and pulmonary embolism (Miura & Gamblin, 2015).

Some of these adverse events can be minimized or prevented with appropriate premedication. Our institution routinely administers ondansetron, ceftriaxone, diphenhydramine, and dexamethasone prior to the TACE procedure. Patient-controlled analgesia (PCA) is used after the procedure for pain control, with the goal of switching the patient to oral medications prior to discharge.

## IMPLICATIONS FOR ADVANCED PRACTITIONERS

When HCC is confined to the liver, it can be successfully treated with DEB-TACE. Current recommendations have expanded the use of TACE to patients with more advanced disease, including those with BCLC C disease and a good performance status and liver function. Patients with HCC rarely present without underlying liver disease, which poses its own mortality risk and can affect patients’ ability to tolerate the TACE procedure. We do not perform TACE on patients with an ECOG PS > 2. Patients with encephalopathy and poorly controlled ascites related to underlying liver disease are not candidates for TACE at our institution.

The TACE procedure is also being evaluated in combination with sorafenib, where ischemic damage caused by TACE and the subsequent release of angiogenic growth factors may be addressed by use of this kinase inhibitor.

Chemoembolization is considered palliative therapy. Although a cure for unresectable HCC is possible after TACE, it rarely occurs. Repeated segmental selective TACE tailored to tumor response has been shown to significantly improve survival in patients with unresectable HCC (Dhansekaran et al., 2010; Song et al., 2012).

The rationale for TACE in our patient (THA) was to provide bridging therapy prior to liver transplantation. It provided a means to control tumor growth while the patient awaited organ allocation and resulted in tumor necrosis, which had the added benefit of reducing the risk for tumor dissemination during transplant surgery. Approximately 20% of patients drop off the transplant list due to tumor progression (Durham & Ray, 2012). The TACE procedure can also provide an interval in which aggressive tumor biology may manifest (Vogl et al., 2009).

## CONCLUSION

Overall, chemoembolization provides localized therapy to isolated malignant lesions. Adverse events due to the chemotherapy are limited, and the procedure can be performed with a minimal hospital stay. Treatment can provide benefit to patients by keeping the tumor in check, either for palliation or as a bridge to transplantation. We look forward to the addition of other agents, such as tyrosine kinase inhibitors, to this treatment to further control tumor growth and spread.
